# Preimplantation genetic testing for aneuploidy in young women with recurrent spontaneous abortion

**DOI:** 10.1097/MD.0000000000044361

**Published:** 2025-11-21

**Authors:** Zhixiong Pan, Qiuyan Huang, Ni Tang, Hui Tang, Zhuo Liang, Pinxiu Huang

**Affiliations:** a Clinical Experiment Center, The First Affiliated Hospital of Guilin Medical University, Guiling, China; b Center of Reproductive Medicine, Affiliated Hospital of Youjiang Medical University for Nationalities, Baise, Guangxi, China; c Key Laboratory of Clinical Diagnosis and Treatment Research of High Incidence Disease in Guangxi, Guangxi, China; d Center of Reproductive Medicine, Guangzhou Women and Children’s Medical Center-Liuzhou Hospital, Liuzhou, Guangxi, China; e Center of Reproductive Medicine, The First Affiliated Hospital of Guilin Medical University, Guiling, China.

**Keywords:** in vitro fertilization-embryo transfer (IVF-ET), preimplantation genetic testing for aneuploidy (PGT-A), recurrent abortion, recurrent spontaneous abortion (RSA)

## Abstract

This study aimed to investigate whether preimplantation genetic testing for aneuploidy (PGT-A) improves pregnancy outcomes in in vitro fertilization-embryo transfer (IVF-ET) cycles in patients with recurrent spontaneous abortion (RSA). A total of 216 young patients with RSA (aged <38 years) who underwent IVF were included in this study. Of these, 114 patients opted for PGT-A, comprising 64 patients with a history of embryonic chromosome abnormality (ECA) (Group A) and 50 patients without ECA (Group B). The remaining 102 patients who did not undergo PGT-A served as the Control group, including 51 patients with a history of ECA (Control A) and 51 patients without ECA (Control B). Clinical outcomes were compared between the groups. The live birth rate in Group A was significantly higher compared to Control A (*P* <.05), whereas no significant difference was observed between groups B and Control B (*P* >.05). PGT-A significantly improved clinical outcomes in RSA patients with a history of ECA, but did not show a similar benefit in those without ECA. However, owing to the retrospective nature of the analysis and potential for unmeasured confounding factors, the value of PGT-A in women with RSA warrants further investigation.

## 1. Introduction

Recurrent spontaneous abortion is defined as 2 or more spontaneous pregnancies in women with the same partner. Chromosomal abnormalities in embryos account for approximately 50% of the early pregnancy losses, with numerical chromosomal abnormalities being the most prevalent. Among them, aneuploidy is the most common chromosomal abnormality.^[1–3]^ Preimplantation genetic testing for aneuploidy (PGT-A) enables identification and exclusion of aneuploid embryos. As a recommended clinical screening tool for patients of advanced maternal age, recurrent pregnancy loss, or repeated implantation failure, PGT-A has the potential to reduce the risk of implantation failure or pregnancy loss attributable to embryo aneuploidy.^[1–4]^

Some studies have reported that PGT-A can enhance pregnancy and live birth rate (LBR)s in patients with RSA,^[5,6]^ while others have found no significant improvement in pregnancy outcomes in this population.^[7]^ PGT-A was reported to be without advantage in patients with unexplained recurrent pregnancy loss. Whose embryonic karyotype had not been analyzed.^[7]^ Additionally, patients with RSA experience a miscarriage rate of 14% following embryo transfer, which may be attributed to embryo damage during biopsy or other unidentified factors. Considering these findings, the efficacy of PGT-A in patients with RSA remains a subject of debate and requires further investigation.

To further evaluate the potential benefits of PGT-A in patients with RSA, this study examined the outcomes of PGT-A in patients with RSA, with and without a history of embryonic chromosome abnormality (ECA). By comparing these groups, this study aimed to clarify the clinical utility of PGT-A and to provide evidence-based data to support its use in this patient population.

## 2. Objects and methods

### 2.1. Research object

This was a retrospective study. The participants were patients who underwent assisted reproductive technology at the Reproductive Center of Guangzhou Women and Children’s Medical, Central Liuzhou Hospital between 2019 and 2022. The inclusion criteria were as follows: history of 2 or more spontaneous pregnancies, maternal age <38 years, and normal karyotypes in both partners. Exclusion criteria included abnormal immunological findings in females, prethrombotic state, anatomical abnormalities of the reproductive tract, endocrine disorders, abnormal semen parameters, genital tract infections, and high-risk environmental factors associated with abortion.

**Figure 1.**
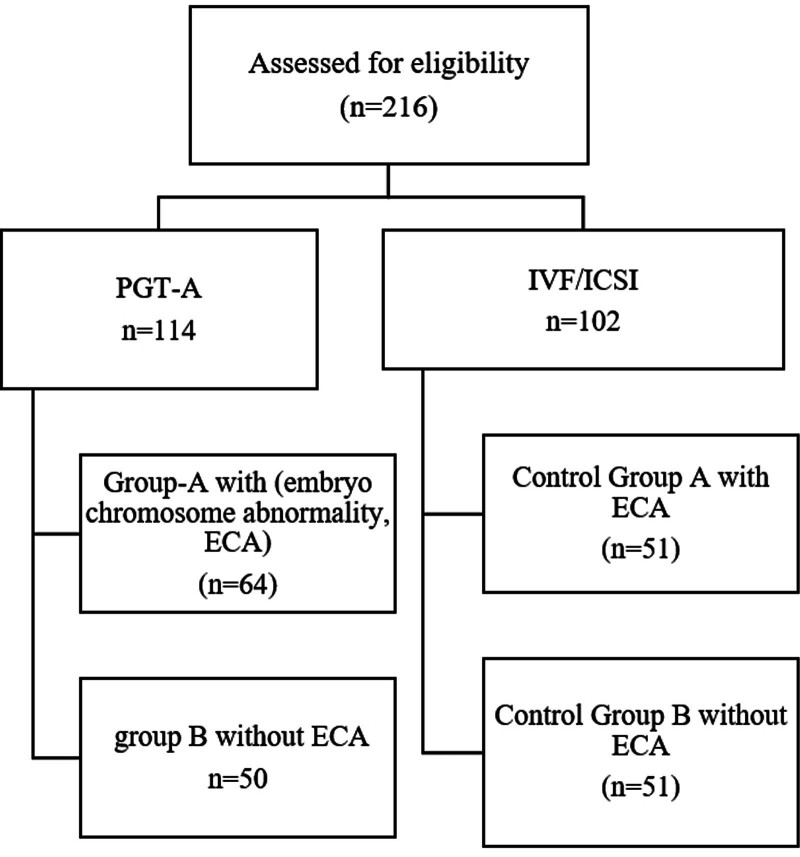
Flow chart of the grouping diagram.

A total of 216 patients were enrolled in the study and categorized into 4 groups: Group A (64 cases) comprised patients who opted for PGT-A with a history of ECA; Group B (50 cases) included patients who chose PGT-A without a history of ECA; and the control group (102 cases) consisted of IVF/ICSI patients who did not undergo PGT-A. The Control group was further divided into Control A (51 patients with a history of ECA) and Control B (51 patients without ECA) (Fig. 1). General demographic and clinical data, including age, baseline FSH levels, body mass index (BMI), clinical pregnancy rate, abortion rate, and LBR, were compared between groups. Based on previous literature reports, although the sample size of this study is not large, it is enough to clarify the problem.

This study was approved by the Medical Ethics Committee, and all patients provided written informed consent prior to treatment.

### 2.2. Methods

#### 2.2.1. *Methods of ovulation promotion and fertilization*

An antagonist ovulation induction protocol was used for ovarian stimulation. human chorionic gonadotropin was administered intramuscularly when at least 2 dominant follicles reached a diameter of >18 mm, and oocyte retrieval was performed via transvaginal ultrasound-guided aspiration 36 hours later.^[8]^ Fertilization was achieved using ICSI in all PGT-A cycles. Fertilization status was assessed 16 to 18 hours post-insemination, followed by blastocyst culture. Blastocysts were graded using Gardner’s system (expansion 1–6, ICM/TE A-C). High-quality embryos were defined as ≥ stage 3 expansion with both ICM and TE ≥ grade B, where A = excellent morphology, B = good, C = poor. This system optimizes embryo selection for assisted reproductive technology.^[9]^

#### 2.2.2. *PGT-A genetic detection technology*

Blastocyst biopsy cells were transferred into PCR tubes and whole-genome amplification and sequencing were performed using the NextseqCN500 high-throughput sequencing kit (Berry and Kang, China). Raw sequencing data were analyzed using the Ke Yun Data Analysis System (Berry and Kang, China) for bioinformatics processing. Chromosomal ploidy, microdeletions, and microduplications were interpreted at a resolution of 10 Mbp.

#### 2.2.3. Embryo freezing and transplantation

Following biopsy, the blastocysts were cryopreserved using vitrification. Blastocysts with normal.

The genetic results were subsequently thawed and a single blastocyst was transferred to each cycle.

#### 2.2.4. Observation indicators

Clinical pregnancy, abortion, and LBRs were compared between groups. Clinical pregnancy was confirmed 25 days after embryo transfer via B-ultrasound, which revealed the presence of a gestational sac. Pregnancy loss occurring before 12 weeks of gestation was defined as an abortion. The clinical pregnancy rate was calculated as (number of clinical pregnancy cycles/ total number of transfer cycles) × 100%; the abortion rate was determined as (number of abortion cycles/ number of clinical pregnancy cycles) × 100%, and the LBR was calculated as (number of live birth cycles/ total number of transfer cycles) × 100%.

#### 2.2.5. Statistical analysis

Analysis was carried out using SPSS software (version 13.0, Chicago), the measurement data were expressed as x ± s, and the average of more than 3 groups was analyzed by variance. Percentage comparisons were performed using the χ2 test. Statistical significance was set at *P* <.05. Binary logistic regression analysis was used to analyze the correlation between PGT-A and LBR. Results were presented as odds ratios (OR) with corresponding 95% confidence intervals. *P*-values <.05 were considered statistically significant.

## 3. Results

No statistically significant differences were observed in age, baseline FSH levels, BMI, or other general characteristics among the 4 groups (Table 1).

**Table 1:** To compare the general data of the recurrent spontaneous abortion patients in each group.

Variables	PGT-A group A (n = 64)	Control group A (n = 51)	PGT-A group B (n = 50)	Control group B (n = 51)	*P*-value
Age (year)	34.32 ± 6.52	34.55 ± 4.47	34.34 ± 4.52	34.35 ± 6.35	.324
Basis FSH (IU/L)	6.44 ± 3.43	6.63 ± 3.21	6.46 ± 1.45	6.71 ± 3.17	.235
AMH (ng/mL)	3.14 ± 1.13	3.33 ± 2.43	3.16 ± 3.33	3.21 ± 2.19	.515
BMI (kg/m^2^)	20.52 ± 2.43	21.42 ± 3.02	20.55 ± 2.07	21.38 ± 2.56	.129
Number of previous abortions (number)	3.55 ± 1.45	3.61 ± 1.63	3.62 ± 1.61	3.56 ± 1.54	.072

PGT-A = preimplantation genetic testing for aneuploidy.

Measurement data was expressed as Mean ± standard deviation.

The clinical pregnancy rates in Group A, Control A, Group B, and Control B were 60.93%, 39.22%, 44.00%, and 41.18%, respectively. Similarly, the LBRs in these groups were 54.69%, 33.33%, 38.00%, and 35.29%, respectively (Table 2). The clinical pregnancy and LBRs in Group A significantly higher than those in Control A (*P* <.05), whereas no significant differences were observed between Groups B and Control B. The abortion rates for the 4 groups were 10.26%, 19.05%, 13.63%, and 14.28%, respectively. Although no statistically significant differences were found among the groups, the abortion rate in Group A was the lowest.

**Table 2:** To compare the clinical outcomes each group.

Variables	PGT-A group A (n = 64)	Control group A (n = 51)	PGT-A group B (n = 50)	Control Group B (n = 51)	*P*–value
Endometrial thickness on hCG day (mm)	10.02 ± 2.54	10.45 ± 2.97	10.15 ± 2.92	10.53 ± 3.06	.415
ET blastocyst number	1	1	1	1	–
High quality blastocyst rate	62.50% (40/64)	60.78% (31/51)	62.00% (31/50)	60.78% (31/51)	.069
Clinical pregnancy rate (%)	60.93% (39/64)*	39.22% (21/51)	44.00% (22/50)	41.18% (21/51)	.035
abortion rates (%)	10.26% (4/39)	19.05% (4/21)	13.63% (3/22)	14.28% (3/21)	.094
Live birth rate (%)	54.69% (35/64)*	33.33% (17/51)	38.00% (19/50)	35.29% (18/51)	.022

ET = embryo transplantation, PGT-A = preimplantation genetic testing for aneuploidy.

Categorical data was shown as n (%), and measurement data was expressed as mean ± standard deviation.

* Compared with the control Group A, it increased significantly, *P*<.05.

The OR with the corresponding 95% CI and P values for each parameter was included in the regression model. After adjusting for female age, basic FSH, AMH, BMI, number of previous abortions, endometrial thickness on hCG day, the only significant *P* value (<.008) was group A with (ECA and PGT) (OR = 1.233, 95%CI: [1.235; 2.245]), the other groups had no effect on the LBR (Table 3).

**Table 3:** Binary regression model.

	Significance	EXP (B)	The 95% confidence interval for the EXP (B)	
			Lower limits	Upper limit
Basic FSH	0.082	0.992	0.984	1.001
Age	0.058	0.903	0.956	2.909
AMH	0.065	0.866	0.752	1.397
BMI	0.078	0.963	0.950	1.276
Number of previous abortions	0.091	1.008	0.805	1.011
Endometrial thickness on hCG day	0.075	1.059	0.856	1.571
Four different groups				
Without (ECA and PGT)	Reference			
Without ECA but with PGT	0.615	1.023	0.804	1.756
With (ECA and PGT)	0.008	1.233	1.235	2.245
With ECA but without PGT	0.856	0.965	0.756	1.846

BMI = body mass index, ECA = embryonic chromosome abnormality, PGT = preimplantation genetic testing.

## 4. Discussion

Aneuploidy is a significant factor contributing to RSA. As a prenatal diagnostic tool, PGT-A is increasingly being used in assisted reproduction because of its ability to screen for chromosomal abnormalities. However, its effectiveness in patients with RSA, particularly young women, remains controversial, raising questions regarding its appropriate clinical application.

Some studies have supported the use of PGT-A in RSA. For instance, Sato et al demonstrated that PGT-A improves LBRs and reduces miscarriage rates in RSA patients with embryonic chromosomal abnormalities.^[10]^ Similarly, Pantou et al highlighted the benefits of PGT-A in specific groups such as RSA or recurrent implantation failure.^[11]^ However, some studies have questioned the efficacy of this treatment. Mastenbroek et al found that PGT-A did not increase clinical pregnancy rates and even suggested a potential decline, challenging its value.^[12]^ Murugappan et al also reported no improvement in pregnancy rates or reduction in miscarriage rates with PGT-A compared with conventional IVF.^[13]^ Yan et al further indicated that conventional IVF yielded non-inferior cumulative LBRs compared to PGT-A in women aged 20 to 37 years with multiple good-quality blastocysts.^[14]^ Therefore, the effectiveness of PGT-A in RSA remains debatable.^[15,16]^

PGT-A is most effective in patients with prior embryo chromosome abnormalities (ECA) because these patients have demonstrated a predisposition to producing aneuploid embryos, making embryo selection crucial. By identifying and transferring only euploid embryos, PGT-A directly addresses the primary cause of failure in these cases – chromosomal abnormalities – thereby improving implantation rates and reducing miscarriage risk while avoiding futile transfers. In contrast, patients without prior ECA often have infertility causes unrelated to aneuploidy (e.g., uterine factors or sperm DNA fragmentation), making PGT-A less impactful. Additionally, younger patients or those with no history of ECA typically produce more euploid embryos, reducing PGT-A’s relative benefit. Thus, PGT-A’s value is clearest in ECA cases where aneuploidy is the main barrier to success, whereas its utility in other populations remains uncertain without clear evidence of chromosomal issues.^[14]^ Proper patient selection is key – PGT-A should be strongly considered for those with recurrent aneuploidy-related failures but used more cautiously in others.

To clarify its role, this study excluded factors such as advanced age, immune abnormalities, and thrombotic states, focusing on patients with RSA, with or without a history of embryonic chromosomal abnormalities (ECA). The results showed that PGT-A significantly improved LBRs in RSA patients with a history of ECA (Group A) compared to the control group, but no significant benefit was observed in RSA patients without ECA (Group B). This suggests that PGT-A is beneficial only in patients with RSA and a history of ECA. However, the immune microenvironment of the endometrium, which may play a critical role in RSA, has not been assessed, and the cause of RSA in patients without ECA remains unclear.^[17,18]^ The immune microenvironment of the endometrium is critically regulated by uterine NK cells (uNK), regulatory T cells (Tregs), macrophages, dendritic cells, and key cytokines including IL-10, TGF-β, and IFN-γ. These components work in concert to establish the delicate balance between immune tolerance and defense required for successful embryo implantation during the window of receptivity. Currently, there is still no unified standard for diagnosing endometrial immune disorders.^[18]^

PGT-A offers psychological and financial benefits for patients with recurrent ECA by reducing miscarriage trauma and futile cycles. However, in low-resource settings, its high cost, infrastructure demands, and risk of embryo misdiagnosis may outweigh advantages. Careful patient selection and counseling are crucial to prevent financial strain and false hopes, as indiscriminate use could worsen healthcare disparities. The test’s value depends heavily on local resources and individual clinical contexts.

The limitations of this study include its retrospective design, small sample size, and unmeasured confounders such as endometrial immune status. Although PGT-A shows promise for RSA patients with ECA, its broader value remains unclear. Given the psychological and financial burdens associated with PGT-A, patients should be thoroughly informed of its potential benefits and limitations before opting for it.

## Author contributions

**Conception and design:** Hui Tang, Zhuo Liang.

**Writing – original draft:** Qiuyan Huang, Zhixiong Pan, Ni Tang.

**Administrative support:** Pinxiu Huang.

**Supervision:** Pinxiu Huang.
